# A consensus statement on detection of hippocampal sharp wave ripples and differentiation from other fast oscillations

**DOI:** 10.1038/s41467-022-33536-x

**Published:** 2022-10-12

**Authors:** Anli A. Liu, Simon Henin, Saman Abbaspoor, Anatol Bragin, Elizabeth A. Buffalo, Jordan S. Farrell, David J. Foster, Loren M. Frank, Tamara Gedankien, Jean Gotman, Jennifer A. Guidera, Kari L. Hoffman, Joshua Jacobs, Michael J. Kahana, Lin Li, Zhenrui Liao, Jack J. Lin, Attila Losonczy, Rafael Malach, Matthijs A. van der Meer, Kathryn McClain, Bruce L. McNaughton, Yitzhak Norman, Andrea Navas-Olive, Liset M. de la Prida, Jon W. Rueckemann, John J. Sakon, Ivan Skelin, Ivan Soltesz, Bernhard P. Staresina, Shennan A. Weiss, Matthew A. Wilson, Kareem A. Zaghloul, Michaël Zugaro, György Buzsáki

**Affiliations:** 1 Department of Neurology, NYU Grossman School of Medicine, New York, NY USA; 2 Neuroscience Institute, NYU Langone Medical Center, New York, NY USA; 3 Department of Psychology, Vanderbilt University, Nashville, TN USA; 4 Department of Neurology, David Geffen School of Medicine at UCLA, Los Angeles, CA USA; 5 Department of Physiology and Biophysics, Washington National Primate Center, University of Washington, Seattle, WA USA; 6 Department of Neurosurgery, Stanford University, Stanford, CA USA; 7 Department of Psychology and Helen Wills Neuroscience Institute, University of California, Berkeley, Berkeley, CA USA; 8 Kavli Institute for Fundamental Neuroscience, Center for Integrative Neuroscience and Department of Physiology, University of California San Francisco, San Francisco, CA USA; 9 Howard Hughes Medical Institute, Chevy Chase, MD USA; 10 Department of Biomedical Engineering, Department of Neurological Surgery, Columbia University, New York, NY USA; 11 Montreal Neurological Institute, McGill University, Montreal, QC Canada; 12 Medical Scientist Training Program, Department of Bioengineering, University of California, San Francisco, San Francisco, CA USA; 13 Vanderbilt Brain Institute, Vanderbilt University, Nashville, TN USA; 14 Department of Psychology, University of Pennsylvania, Philadelphia, PA USA; 15 Department of Biomedical Engineering, University of North Texas, Denton, TX USA; 16 Department of Neuroscience, Columbia University, New York, NY USA; 17 Department of Neurology, Center for Mind and Brain, University of California Davis, Oakland, CA USA; 18 Department of Brain Sciences, Weizmann Institute of Science, Rehovot, Israel; 19 Department of Psychological and Brain Sciences, Dartmouth College, Hanover, NH USA; 20 The Canadian Centre for Behavioural Neuroscience, University of Lethbridge, Lethbridge, AB Canada; 21 Department of Neurological Surgery, University of California, San Francisco, CA USA; 22 Instituto Cajal, CSIC, Madrid, Spain; 23 Department of Experimental Psychology, Oxford Centre for Human Brain Activity, Wellcome Centre for Integrative Neuroimaging, Department of Psychiatry, University of Oxford, Oxford, UK; 24 Brookdale Hospital Medical Center, SUNY Downstate Medical Center, Brooklyn, NY USA; 25 Department of Brain and Cognitive Sciences and Picower Institute for Learning and Memory, Massachusetts Institute of Technology, Cambridge, MA USA; 26 Surgical Neurology Branch, National Institute of Neurological Disorders and Stroke (NINDS), National Institutes of Health, Bethesda, MD USA; 27 Center for Interdisciplinary Research in Biology (CIRB), Collège de France, CNRS, INSERM, Université PSL, Paris, France

**Keywords:** Attention, Decision, Cellular neuroscience

## Abstract

Decades of rodent research have established the role of hippocampal sharp wave ripples (SPW-Rs) in consolidating and guiding experience. More recently, intracranial recordings in humans have suggested their role in episodic and semantic memory. Yet, common standards for recording, detection, and reporting do not exist. Here, we outline the methodological challenges involved in detecting ripple events and offer practical recommendations to improve separation from other high-frequency oscillations. We argue that shared experimental, detection, and reporting standards will provide a solid foundation for future translational discovery.

## Abstract (web summary)

While the contribution of sharp wave ripples in memory consolidation and decision-making is established in rodent models, our understanding of their role in human memory is incomplete. Here, the authors discuss common methodological challenges in detecting, analyzing, and reporting sharp wave ripples, then they suggest practical solutions to distinguish them from other high-frequency events

## Introduction

Interest in hippocampal sharp wave ripples (SPW-Rs) has accelerated over the past decade. SPW-Rs are highly conserved among mammals, but their presence in lizards and birds has been debated^1,2^. Their necessity for memory consolidation and working memory has been demonstrated through disrupting or altering their duration^3–7^. SPW-Rs are the most synchronous pattern in the mammalian brain^8^, exerting a widespread impact on neocortical and subcortical structures^9–12^. Activity during SPW-Rs represents compressed forward and reverse population spike sequences, in which past experience is replayed^13–15^ and flexibly recombined to depict potential future scenarios^13,16,17^. During awake states, this internal generation of possible options contributes to the selection of an optimal strategy without requiring physical exploration (but see ref. 18). Reshuffling of newly acquired and existing knowledge supports generalization, abstraction, and creative thought^19–24^.

**Fig. 1:**
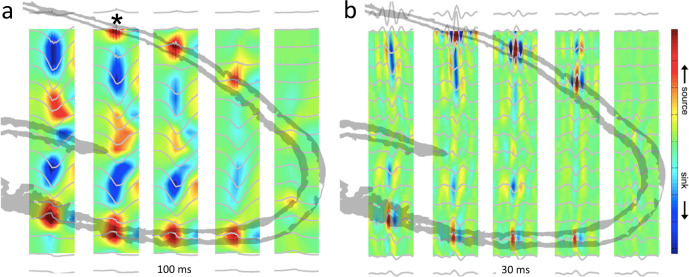
Depth profile of SPW-Rs in the hippocampal CA1-dentate axis. **a** Recording with a 6-shank, 96-site linear silicon probe spanning hippocampal regions and layers in a rat (5-shanks are shown, each with 16 sites with 100 µm vertical separation). Average current source density (CSD, color) maps and superimposed LFP traces of SPW-R events (100 ms, gray) from all recording sites. Asterisk indicates reference site. Note negative sharp waves and sinks (blue) in the stratum radiatum of CA1 and CA3 and the inner molecular layer of the dentate gyrus. **b** Same as in (**a**) but the maps were constructed from the filtered signal (50–250 Hz; 30 ms long traces). Red, source; blue, sink. Reproduced from ref. 25, CC BY-NC-SA 3.0 (https://creativecommons.org/licenses/by-nc-sa/3.0/).

**Fig. 2:**
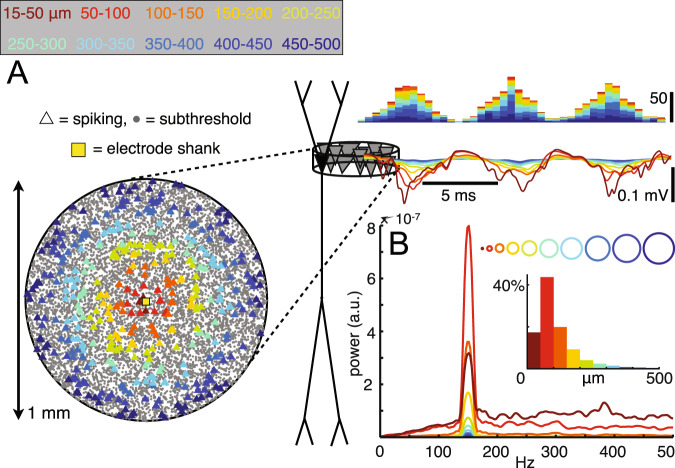
Spikes from groups of distant neurons contribute substantially to fast LFP oscillations. **A** Histograms of extracellular spikes (top right) extracellular voltages along the CA1 stratum oriens–stratum radiatum axis in a rhythmically bursting population with ~6% of the population firing in each 10 ms interval. Spike bursts recur periodically at 150 Hz and have a Gaussian shape. The locations of neurons that spike during one 6.7 ms ripple period are indicated by triangles in a top-down view of the pyramidal layer (left), with colors indicating the 50µm-wide ring from which the spikes originate. Voltage traces are colored correspondingly, with contributions from each ring of cells adding cumulatively from the outside in. The colors in the histograms and current traces correspond to the cumulative contribution of the neurons in the ring. **B** Averaged power spectra of the CA1 stratum pyramidale traces from each individual ring. The insets indicate the proportions of the total voltage power at 150 Hz generated by each ring- or disk-shaped subpopulation (i.e., the peak values of the power spectra, normalized by the power at 150 Hz in the full population). Reproduced from ref. 32, CC BY-NC-SA 3.0 (https://creativecommons.org/licenses/by-nc-sa/3.0/).

The hippocampal SPW-R is a complex LFP pattern of two interdependent but temporally related events (Fig. 1). The extracellular *sharp wave* (SPW) is produced by large transmembrane currents in the apical dendrites of CA1 pyramidal cells, which are triggered by synchronous CA3 input targeting the mid stratum radiatum^25,26^. This CA3 volley also excites CA1 interneurons to protract the rate of pyramidal neuron recruitment. Their interaction induces a brief oscillation, detected as a “ripple” (110–180 Hz in rodents) in the LFP^27–30^. The LFP *ripple* is composed of positive ‘domes’, reflecting perisomatic fast inhibitory currents in pyramidal neuron, and sharp negative troughs, reflecting synchronous spikes (‘mini population spikes’)^31,32^, respectively (Fig. 2). Rare deviations from this general pattern occur when CA2 pyramidal neurons induce negative SPWs in CA1 str. oriens^33^.

Recently, several groups have demonstrated the role of putative human SPW-Rs in episodic memory using high-density recordings, intracranial EEG and magnetoencephalography (MEG) recordings^34–39^. These studies suggest a translational link to decades of rodent work. At this pivotal moment in scientific discovery, we are confronted with a lack of consensus on recording, detection, and reporting methods for SPW-Rs. Methods vary from paper to paper (Supplementary Table S1 and see ref. 40), which likely drives much of the variance across laboratories. To discuss these challenges, we gathered more than 30 neuroscientists actively studying hippocampal SPW-Rs in rodents, non-human primates, and humans. The group agreed on the necessity of establishing common experimental, detection and reporting standards as a foundation for translational work. Below, we discuss several problems and make recommendations for future investigation in SPW-Rs.

## Problem 1: Combating artifacts

In experimental animals, SPW-Rs can be confidently measured with linear electrode arrays, as the sharp wave and ripple components are simultaneously recorded across CA1 layers (str. oriens, pyramidal layer and str. radiatum). The current source-density (CSD) profiles of both events can be identified by automated detection thresholds, then visually inspected by expert operators^5^. Recording across layers facilitates rejection of artifacts, which are observed as power increases across simultaneously recorded sites. These curated data are considered to be “ground truth,” useful for training and testing detection algorithms (https://github.com/buzsakilab/buzcode/blob/master/detectors/detectEvents/detect_swr/detect_swr.m). Deviating from this ideal scenario, reliable identification of SPW-Rs in humans with sparse electrode coverage introduces daunting challenges.

Most physiological phenomena belong to a continuum. In practice, we use arbitrarily defined boundaries to group events and to study their physiological and behavioral roles. We often parse the LFP by frequency bands. In rodents, a bandpass filter of 120–160 Hz is often used for ripple detection because their dominant frequency falls within this range. Unfortunately, filtering often distorts waveforms and may produce fast oscillation-like patterns even from single transients.

### Non-biological noise

Electric noise from laboratory equipment (e.g., centrifuges, refrigerators, ventilators, coagulators) can contaminate recordings, especially in the operating room. Filtered line noise can resemble ripple band power. These electrical and radio frequency artifacts can be reduced with a lightweight wire mesh shield (i.e., a Faraday cage) on the head of the animal^41,42^. Applying a duration threshold for ripple detection (e.g., >10 ms) may further attenuate brief environmental artifacts.

### Muscle artifacts

Muscle contractions (i.e., electromyogram, EMG) are the dominant source of biological noise. Muscle contractions generate electric fields that are superimposed on neuronal LFP recordings. EMG artifacts can occur in the ambulating animal as well during drinking, chewing, whisking, teeth chattering and isolated muscle twitches. Head-fixed preparation may amplify EMG artifacts, since animals struggle when they are uncomfortable. In primates, electric fields generated by eye or tongue movements can result in volume-conducted EMG contamination.

Besides muscle contraction, the magnitude of muscle artifact also depends on the spatial relationship between active and reference electrodes. If the EMG field occurs between the active and reference electrodes, greater interelectrode distance results in greater EMG contamination. Placing the reference electrode closer to the hippocampus (e.g., in nearby white matter) may reduce EMG contamination, but potentially distort the LFP waveform, because LFP components recorded by active and reference electrodes will be subtracted. The most effective recording method is to use three (or more) active electrodes spanning across the dipoles formed by SPWs and ripples, referenced to a distal electrode and calculate CSD. This ‘difference of difference’ voltage derivation eliminates far fields and extracts local currents^43^.

Reference electrode placement in humans is limited by clinical constraints. When multiple recording electrodes are used, EMG artifacts are synchronously recorded on most of them. To exclude artifacts during analysis, commonly used methods include re-referencing to the nearest white matter electrode or across all electrodes (average montage). Artifact uniformity can also be exploited by independent component analysis (ICA) or related algorithms^44,45^ and exclude candidate ripples that coincide with the EMG- artifacts detected on the common average^46^. Of note, some of the authors think that recording wideband signals, then eliminating them offline, is preferable to hardware solutions that attenuate online artifacts but distort the recording brain signals in subtle ways^47^. Virtually every hardware method of artifact attenuation can be performed offline and more effectively than online methods.

### Locally recorded spikes

Another common source of false SPW-R detection results from filtering locally recorded action potentials. Larger spikes produce larger artifacts. Filtered non-rhythmic spike bursts and multi-unit bursts are difficult to distinguish from a true ripple oscillation. This is an important issue since the physiological ripple is composed of rhythmic action potentials (Fig. 2). One way to reduce such contamination is to average across multiple recording sites from the same layer (when available^48–50^) or using a neighboring site with less prominent spiking activity.

## Problem 2: Recording and detection of SPW-Rs

### Electrode configuration and layer localization

The gold standard for accurately detecting SPW-Rs comes from high-density laminar sampling of LFP across the CA1 layers (Fig. 1). Multi-laminar recordings can also detect and differentiate rare SPW-R events, for example when the CA2 input induces a sink in the str. oriens and a return source in str. radiatum^33^. With independently movable tetrode assemblies, some electrodes are positioned within CA1 pyramidal layer and other electrodes are placed in the str. radiatum to monitor ripples and SPWs simultaneously. In most situations, the LFP is not recorded with such high spatial resolution. Ripples can be recorded by different types of electrodes, including glass micropipettes^51–54^, multi-site silicon probes^5,29,30,33,52,54,55^, tetrode wire assemblies^14,56–58^, and 50 μm diameter metal wires^59–61^. While a quantitative comparison of the electrode types has not been performed, the size and impedance of the electrode likely biases the volume of contributing neurons monitored by the electrode^43^.

The amplitude of the LFP ripple depends on both synchrony of spiking and the orientation of the ripple current-generating pyramidal neurons. In rodents, high packing density in the pyramidal layer generates a relatively large ripple amplitude. The largest ripples are recorded from the middle of the pyramidal layer; amplitude dwindles with greater distance from the pyramidal layer (Fig. 1^62^). Ripples cannot be reliably detected even a few hundred µm from the pyramidal layer. Because SPW-Rs are rarely synchronous over the entire septotemporal axis, recording from a single hippocampal site does not exclude the possibility that non-propagating, lower amplitude SPW-Rs occur at other, non-recorded sites^63^. In humans and non-human primates, neurons in the CA1 pyramidal layer are scattered over several hundred micrometers^64^, thus the biophysical events may be somewhat different, while the essential characteristics of ripples localized to the CA1 pyramidal layer appears to be well conserved^94,95^.

**Fig. 3:**
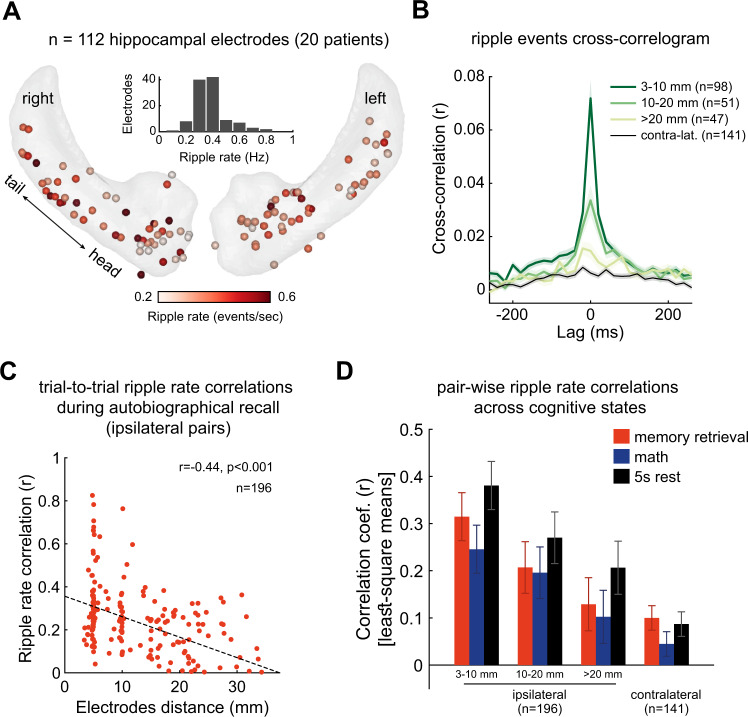
Trial-to-trial SPW-R rate correlations within and across the hemispheres. **A** SPW-R rates computed throughout the entire task (20 patients). Inset: distribution of SPW-R rates across electrodes. **B** Coincident activation of SPW-Rs as a function of electrode (0.86 mm in diameter) distance (intercontact distance = 4–5 mm). **C** Trial-to-trail correlation between SPW-R rates during memory retrieval trials. **D** Correlation coefficients between the contralateral sites were much weaker than across electrode pairs in the same hippocampus/subiculum. Reproduced from ref. 37 with permission, Elsevier.

While both SPWs and ripple events in rodents often occur in both hippocampi synchronously and symmetrically, the phase of the ripple cycle is randomly aligned^65,66^. In humans, ripple emission is rarely synchronized across the two hemispheres and often spatially confined within the same hemisphere^37,67^ (Fig. 3). The reduced bilateral synchrony may be due to the diminutive ventral hippocampal commissure in primates^67^.

**Fig. 4:**
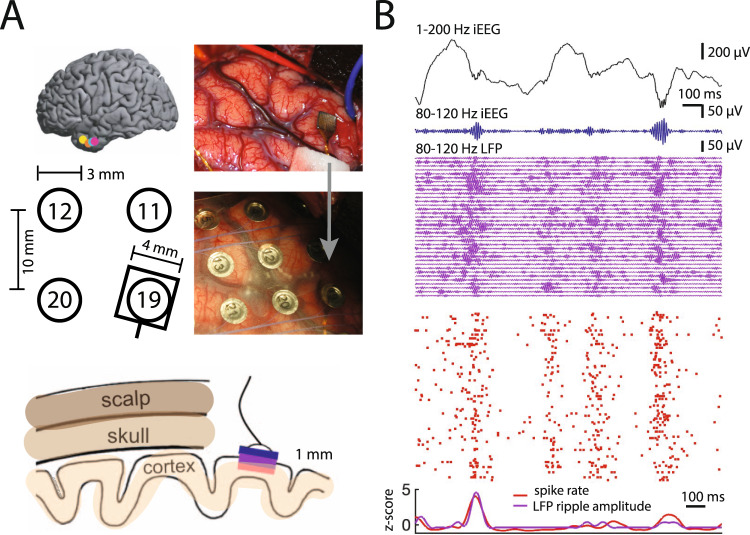
Relationship between cortical ripple amplitude and local spiking. **A** Locations of the microelectrode arrays with respect to four nearby iEEG channels in one participant (bottom left). Right, Intraoperative photo of implanted array in the anterior temporal lobe before and after placement of an iEEG grid over the it. Bottom, Schematic of scalp, skull and cortex with respect to one iEEG channel on the cortical surface and one array in cortex. **B** 1500 ms window of 1–200 Hz iEEG signal (black), 80–120 Hz band iEEG signal (blue), 80–120 Hz band LFP signals across all MEA electrodes (purple), and raster plot for sorted units (red). Reprinted from ref. 73.

As SPW-R are only recorded in the immediate vicinity of the CA1 pyramidal layer in rodents and monkeys, macroelectrode detection of ripples in human hippocampus is surprising. Several papers have reported electrode placement within hippocampus, or specified subfield-level locations (e.g., dentate, CA1-CA3)^36,37,39,68^. However, localization by layer is rare^9,12,69–72^ and the recording sites often include mixed hippocampal, subicular and entorhinal regions. Such variability in electrode location between rodents and humans makes direct comparison challenging. For such comparison to be feasible, future intracranial studies in humans should determine electrode localization by layer and confirm that the ripples detected in the intracranial EEG (iEEG) macroelectrodes originate from spiking activity in the pyramidal layer of CA1 (preferably during non-attentive brain states such as rest or NREM sleep). Furthermore, a recent intracranial study that conducted simultaneous recordings of macro- and micro-LFP, with neuronal spiking in the superficial neocortical layer, has demonstrated a relationship between the amplitude and duration of ripple in the micro-scale LFP signals, macro-scale iEEG and neuronal spiking synchrony (Fig. 4)^73^. Similar comparison of verified SPW-Rs recorded with microelectrodes in the CA1 pyramidal layer and macro-scale iEEG signal will be necessary to verify or refute whether fast LFP oscillations in the 80–150 Hz band reflect true SPW-Rs or other fast signals.

Anatomical layer identification is critical because gamma activity from the low (30 Hz) to high (150 Hz) sub-bands is observed in the dendritic layers, representing “projected patterns” (i.e., induced transmembrane currents in the target dendrites) from upstream regions^74–77^. For example, theta phase-locked gamma patterns (100–150 Hz) and entrained granule cell spikes are prominent in the dentate molecular layer, projected from layer 2 neurons of the medial entorhinal cortex^75,78^. Distinguishing between CA1 pyramidal layer (ripple) and dendritic layer (gamma) patterns should be a high priority for future experiments (see Problem 6). If layer localization is not possible by imaging and macro-microelectrodes, spectral components of the LFP surrounding the high-frequency oscillation pattern may be useful (see below).

### Arbitrary detection thresholds result in variable SPW-R rates

Even when electrodes are confidently located in the CA1 pyramidal layer, frequency band, duration, and amplitude thresholds for detecting hippocampal SPW-Rs vary widely across rodent, non-human primate, and human laboratories (Supplementary Table S1). Detection parameters can vary within the same laboratory. The morphological features of SPW-Rs exist on a continuum that reflects the activity and interactions among the contributing neurons. These features are discretized by the experimenter using arbitrary thresholds.

**Fig. 5:**
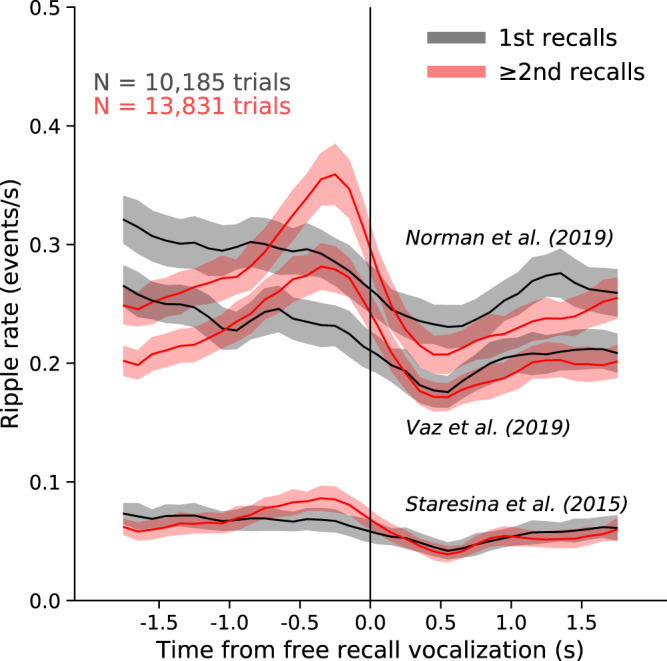
SPW-Rs aligned to verbal recall for three different detection methods. Human intracranial hippocampal CA1 recordings were taken while patients (*n* = 96) performed a free recall task from a 12-word list (from ref. 109). Recalls were split into the first recall and the remaining (≥2nd) recalls from each list. Ripples were detected using three different published methods (refs. 34, 36, 111) and peri-vocalization time histograms were averaged across trials pooled for all patients using 100 ms bins and a 5-point triangle smooth. While the rise in ripples before recall vocalization for ≥2nd recalls compared to 1st recalls is statistically different for all three detectors, the detected ripple rates vary several-fold depending on the detection method. Figure courtesy of John Sakon.

SPW-R frequency band criterion for rodents (100 to 250 Hz) is generally higher than for monkeys (95 to 250 Hz) or humans (70–250 Hz, most use 80–150 Hz bandpass filters; Supplementary Table S1). However, the use of arbitrary voltage thresholds or even standard deviations relative to background activity make normative values experiment-specific. The amplitude threshold of the integrated ripple power varies from 2 to 7 standard deviations from the background activity in various papers. Unfortunately, because the calculation of standard deviation is performed against background activity, the detected incidence of ripple events is inevitably influenced by brain state changes. Variable duration thresholds (>10 ms) greatly influence the reported incidence of SPW-Rs (Fig. 5). Thus, reported values can vary two orders of magnitude across studies (from 0.01 to >10 Hz; for durations from 10 to >100 ms (Supplementary Table S1).

Furthermore, the inclusion of the sharp wave component influences detection rates. A recent study reported that waking ripple density as 1.9 events/min if a sharp wave was included among the ripple detection criteria^72^. Another study reported 10–40 events/min when a sharp wave was not required^36^. Another source of variability of SPW-R rates are different arousal states across experiments. One way to reduce inter-study and inter-species variability is to use SPW-R rates recorded during NREM sleep as a benchmark.

Lowering detection thresholds for voltage or duration increases the detected ripple rate but increases the likelihood of false-positive events. To reduce the rate of false-positives, automated detections should be visually inspected by an expert. Conversely, human subjectivity and memory bias are important confounds and also cannot be used alone. Besides visual inspection, auditory evaluation of putative SPW-Rs can be useful because the ear is a natural Fourier analyzer^79^. Finally, because real ripple power and duration follow log-normal distributions^80^, the log-distribution of detected events should be reported (instead of mean values).

### Data-driven, automated approaches to SPW-R detection

Instead of relying on arbitrary thresholds, several laboratories have developed automated detection methods^8,81^. For example, the distribution of ripple frequency events exceeding 20 ms can modeled against background ‘noise’ and the termination of the SPW-R envelope occurs with return to the session mean^81^. Yet, even this method is inadequate if quantitative SPW-R counts are compared across sleep and wake because these states have different background LFP power.

Supervised machine learning approaches, such as Recurrent Neural Networks (RNN) with Long Short-Term memory (LSTM) layers or Convolutional Neural Networks (CNN), can learn from curated datasets to recognize distinct features of SPW-R events^82,83^. Alternatively, unsupervised techniques can separate SPW-R from pathological events^84,85^. Automated approaches are advantageous because of their objectivity and consistency. Parameters of the automated detection can be precisely defined and communicated, in contrast to the subjective judgments of human operators. Several automated programs have been developed for the detection of fast frequency oscillations for clinical use^86–88^, although their performance on SPW-R detection needs to be evaluated.

The problem of reliable SPW-R detection is amplified in real time applications^89^. Precise, reliable online detection of SPW-R events is critical for the development of closed-loop perturbations. Improvement is especially critical for interventions when interruption of SPW-Rs is the goal and identification of SPW-Rs is based on a short ripple fragment (typically 2 to 4 cycles and possibly associated SPW)^5,90^. In humans, where electrodes are difficult to precisely locate to the hippocampal subfield layer, a simple bandpass filter for closed-loop experiments would not suffice. With filter-based methods only approximately half of the larger amplitude ripples are detected^47,89^. Thus, novel strategies that improve detection quality are needed^40,83,91^. For some applications, detecting neuronal sequences, as opposed to LFP features, may be more reliable^92,93^, although online detection also faces the problem of short fragments. The development of a publicly available ripple detection algorithm, tested by the community (perhaps dedicated platforms for rodents and humans, depending on constraints), is highly desirable^40,89^.

## Problem 3. Arousal, attention, and behavioral states

### SPW-Rs dominate low arousal states in rodents and non-human primates

SPW-Rs in mice, rats, bats, rabbits and cats occur during periods of behavioral quiescence, such as pauses in locomotion and NREM sleep. In contrast, theta oscillations dominate active states such as exploration, attentiveness, and REM sleep^94^. This anti-correlation can be attributed to subcortical neuromodulators, especially acetylcholine which tends to be higher during movement^95^. Activation of cholinergic neurons in the medial septum or neurons in locus coeruleus and median raphe promotes theta activity while suppressing SPW-Rs^26,96^. Thus, selecting periods when the animal is immobile (e.g., excluding events that occur at >5 cm/s speed) facilitates the reliable detection of SPW-Rs.

Similarly, SPW-Rs in macaque monkeys occur during rest periods between tasks and grooming^97,98^. As in rodents, ripples are present in and near the CA1 pyramidal layer but not in other layers^98^. The oscillation frequency of ripples in primates is slower (110–125 Hz) than in rodents. Concurrent with ripple occurrence, SPWs are present in the str. radiatum with polarity reversal in the pyramidal layer and str. oriens. Increased power of slow-wave activity during these quiescent periods correlates with bouts of SPW-Rs events especially with eye closure^98^. Thus, SPW-Rs in rodents and monkeys share physiological and behavioral characteristics.

SPW-Rs have also been reported in macaque monkeys during a visual search task^97^. As head-restrained macaques attend to the visual stimulus, SPW-Rs are largely absent, but occur prior to memory retrieval^22,97^. This resembles SPW-R occurrence in rodents during pauses from exploration, and has recently been described in human studies (see below).

Theta patterns in the primate hippocampus occur in short bouts, often locked to eye saccades^99–101^. This may be similar to activity in the rodent ventral hippocampus (corresponding to the uncus and body of the primate hippocampus). Rodent theta in the dorsal hippocampus is prominent during ambulation and REM sleep, but intermittent in the ventral hippocampus^102^. Future investigations in rodents should examine whether SPW-Rs in the ventral hippocampus can emerge during attentive behavior. This distinction may have important physiological implications since SPW-Rs in the dorsal and ventral hippocampus occur largely independently from each other^102,103^.

### Behavioral states in humans and animals are characterized differently

While the behavioral correlates of SPW-Rs in rodents are well characterized, quantitative description of correlations in humans is lacking due to technical constraints or clinical limitations. Except for a few human iEEG studies performed during ambulation^104–106^, cognitive iEEG experiments involve mainly stationary subjects^107^. On the other hand, cognitive states can be inferred in humans through verbal accounts, which is not possible in animal experiments.

In humans, generation of hippocampal SPW-Rs has been reported during memory encoding and retrieval of various stimuli—including visual images to word pairs and face-profession associations^34,36,39^. Human memory retrieval could represent self-generated and spontaneous choice, resembling the rodent choice of trajectory based on past experience^108^. During autobiographical memory recall, human hippocampal ripples correlate with an increase in high-frequency broadband (HFB; 60–160 Hz) activity in the neocortical default mode network (DMN^37^) In episodic and semantic memory tasks, hippocampal ripples occur at a higher probability before successful than failed retrieval^39^, (Fig. 5)^37,109,110^. Of note, ripple rates have been reported to be higher with recall of remote autobiographical or imagined future events, compared to semantic information^37,68^. Conversely, SPW-R rate is decreased during arithmetic calculations^37^, similar to SPW-R suppression in rodents during high attention states^7,20^.

Yet, differences between the relationship between SPW-Rs and behavior in rodents versus primates can be striking. In rodents, SPW-Rs occur several hundred milliseconds to seconds *after* exploration and reward^26^. In macacques, SPW-R occur prior to correct visual memory retrieval. In humans, SPW-Rs have been reported just prior to conscious recall. One possible cause of this difference is the fragmented nature of theta oscillations in primates, which may facilitate emergence of SPW-Rs. Rapid switching between arousal states may allow intermingling of SPW-Rs and theta oscillations. Given the strong SPW-R suppression by subcortical neuromodulators, such as acetylcholine^7,96^, this relationship may imply different dynamics of subcortical neuromodulators in rodents and primates. However, these conjectures need to be tested.

Another possible explanation for the discrepant findings between model systems is that SPW-R and theta states work in succession for effective recall. During retrieval, SPW-Rs may support a pre-conscious search (Fig. 5) by priming neuronal circuits with information drawn from neocortical storage^20^. Conscious recall, which is mentally ‘effortful’, would then be supported by theta/gamma oscillations^112^. This possibility should be carefully examined in future experiments. A final possibility is that putative ‘ripples’ in human studies actually represent other high-frequency events and mechanisms altogether (discussed under Problem 6).

Future human studies should carefully monitor arousal states, particularly during putative ripples. Pupil diameter, heart rate changes and other autonomic features can signify changes in vigilance and attention. Spectral features of the LFP background of detected ripples would confirm arousal state. The large difference within individuals between SPW-R rates during NREM and REM sleep can provide a useful positive control. Waveform, frequency, duration, and amplitude features of NREM SPW-Rs can be compared with supposed ripple events detected during cognitive tasks^68,111^. Assigning likelihood scores to detected SPW-R events from the entire dataset, then performing analysis on a subset of highly likely SPW-Rs, would increase confidence in the findings.

## Problem 4. Ripples observed outside the hippocampal CA1 subfield

Under physiological conditions, ripples are prominent in CA1 because of the strong convergent input from the CA2/3 pyramidal neurons along the septotemporal axis^27,29^. CA1 coupled ripples are present but decrease along the subicular-entorhinal axis^62,66,113^. Naturally-occurring fast oscillations can also be observed in dentate gyrus and CA3 pyramidal layer but they vary across a wide frequency range and their spike content is not phase-locked to the CA1 ripple^25^. Furthermore, when local excitation is augmented by pathology or optogenetic driving of pyramidal neurons, fast oscillations may occur in any hippocampal region or even the neocortex, likely due to the enhanced interaction of fast-spiking perisomatic interneurons and consequent pacing of pyramidal cell spikes^30^.

Besides hippocampus, fast LFP and unit firing oscillations have been described in multiple brain regions in rodents, including lateral septum^10^, amygdala, piriform cortex^114–116^, parietal cortex, and medial neocortex of rodents during NREM sleep or waking rest^117–119^. Recent human investigations have described similar fast oscillations, albeit at a lower frequency (80–120 Hz) in the medial temporal lobe (MTL)^34,36–38,71,109,111,120^ and default network regions of the neocortex, including lateral temporal neocortex, precuneus, and medial prefrontal cortex^34,73,109^. Higher frequency ripples (up to 250 Hz) have been reported in the healthy occipital area^121^. Together, these studies suggest that hippocampal SPW-Rs are coupled to neocortical ripples in memory tasks. Likewise, both events are modulated by neocortical slow oscillations and spindle frequency oscillations during NREM sleep. However, neocortical ripple frequency events should be differentiated from hippocampal SPW-Rs, which are generated by the unique cytoarchitecture of CA3/CA1 subfields.

Source localization of electrical signals requires CSD analysis or, preferably the direct recording of spikes concurrently with LFP. A recent human study^73^ recorded spiking activity and micro-scale LFPs through microelectrode arrays implanted in the lateral temporal cortex (1 mm depth), and simultaneous macro-scale neocortical subdural LFP (3 mm diameter electrodes; iEEG) from medial and anterior temporal lobe as subjects participated in a verbal episodic memory task. The phase of macro-LFP fast oscillations (80–120 Hz) correlated with micro-LFP fast oscillations (80–120 Hz), which in turn correlated to unit firing (Fig. 4^73^). Regardless of whether these transient events are true ripple events, the findings suggest that short-lived fast oscillations recorded even with relatively large surface macroelectrodes can reflect transient bouts of spiking activity in nearby tissue^32^.

Likewise, macro-micro depth electrodes should be used to determine whether detected hippocampal “ripples” are truly SPW-R (with correlated LFP and spike firing) or other high-frequency patterns^119^. Such verification is vital to compare the genesis, localization, state, and behavioral correlates of SPW-Rs in rodents and primates and to explore the possibility that ripple patterns reflect a general feature of neural processing across species and brain regions and species.

## Problem 5. Relationship of SPW-Rs to gamma oscillations and broadband activity

### Gamma oscillations and SPW-R have overlapping frequency bands

Ideally, network patterns should be distinguished by their mechanisms rather than their appearance. Inferring mechanism is challenging from single-site LFP recordings, known as the “inverse problem”^43^. SPW-Rs need to be separated from other high-frequency patterns, such as high-frequency gamma oscillations (high gamma)^122^ and irregular (broadband) high-frequency activity^123,124^. Because fast gamma activity and SPW-Rs possess overlapping frequency bands^20^, their conflation represents an important source of variability across studies and the occasional “contradiction” between rodent and human studies.

An extensive line of work describes the spatiotemporal course of high gamma power and high-frequency activity across the human brain during cognition. Network activity in the 50–140-Hz frequency range increases in power in both neocortex and hippocampus when subjects perform a range of sensorimotor and cognitive tasks. Increased high gamma power for successful vs. unsuccessful memory processes have been reported in a series of iEEG studies^110,112,113,125–128^. Of note, the time course of high gamma power increase is similar to that of increased ripple density, both peaking at ~500–1500 ms after stimulus onset and terminating with memory responses^36,129^. This raises the question of detected SPW-Rs are merely filtered gamma bursts. Conversely, one can argue that broadband gamma activity, typically derived from multiple trials as averaged power over time, consists of multiple ripple events.

### Gamma and SPW-R detection parameters

The inability to measure neuromodulator fluctuations, imprecise electrode localization relative to hippocampal subfields and layers, and rarity of unit-level recordings make the objective separation of hippocampal SPW-Rs from other fast LFP signals in humans difficult. As for SPW-Rs, the magnitude of gamma power is strongly correlated with neuronal spiking^130,131^. Yet, some distinctions between gamma and SPW-Rs can be made even with macroelectrode recordings. While high gamma broadband power typically reflects sustained increases, SPW-Rs are characterized as discrete bursts of high-frequency activity. However, when oscillatory events appear at different frequencies or timepoints across trials, averaging signals across frequencies and timepoints could create the false appearance of a broadband gamma effect. Thus, it is important to distinguish whether apparent periods of gamma power reflect sustained oscillations or bursts of ripples that vary in frequency or latency across trials^73,132^.

### Gamma oscillations and SPW-Rs are generated by different cell types and mechanisms

If both SPW-Rs and gamma oscillations provide synchronous outputs and serve similar functions, is distinction between the two events important? Single neuron-level recordings reveal that the gamma and ripple oscillations are indeed different because they vary with activity from different cell types. For example, chandelier and O-LM interneurons are silent during SPW-Rs but fire synchronously with gamma oscillations^133^. Ripples are confined to the pyramidal layer^29^, while gamma rhythms with current sinks are localized to distinct dendritic layers^76–78,134^. Gamma patterns at all frequencies are phase-locked to the theta cycles^77,78^. In contrast, SPW-Rs are absent during theta but phase-locked to sleep spindles^135^.

**Fig. 6:**
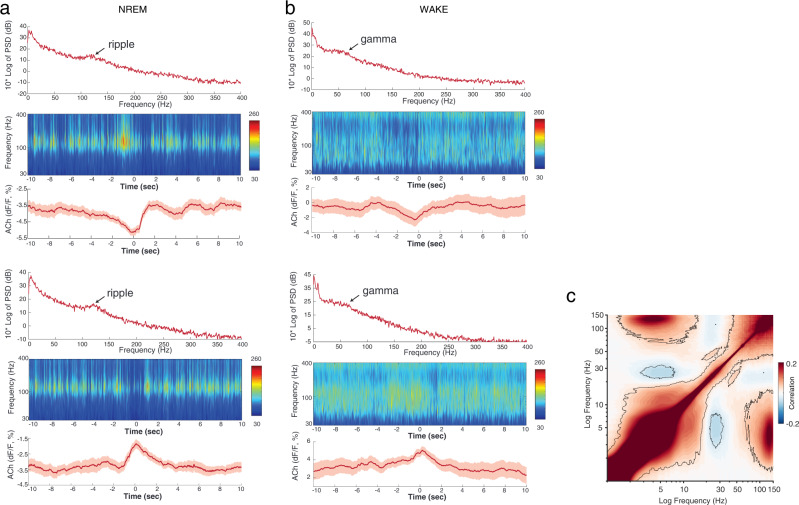
Relationship between Ach levels in the hippocampus and SPW-R/gamma power. **a** Power spectrum (0–400 Hz) and time-resolved power spectrum (40–400 Hz) of the LFP recorded from the CA1 pyramidal layer of a mouse, centered at the natural fluctuations of Ach levels (troughs and peaks, respectively). **b** Same as in **b** but during waking. Note the absence of ripples (>100 Hz) during Ach peaks, whereas highest gamma power (40–120 Hz) is present at the highest levels of Ach release. Note also the different calibration of the power panels and averaged Ach signal. Based on >50 average epochs. **c** Average cross-frequency power comodulogram of the LFP from the CA1 pyramidal layer in a macaque. Note the inverse correlation between ripple and beta/gamma (20–80 Hz) frequency bands. **a**, **b** Reproduced from ref. 7, and **c** reproduced from ref. 140, CC BY-ND 4.0 (https://creativecommons.org/licenses/by-nd/4.0/).

Perhaps the most conspicuous difference between fast gamma and SPW-R oscillations is their anti-correlation with acetylcholine levels. Activation of the basal forebrain cholinergic neurons decreases cholinergic tone^136,137^, associated with elevated SPW-R rate and decreased gamma power. Conversely, optogenetic stimulation of medial septal cholinergic neurons robustly suppresses SPW-Rs^96^ and increases gamma frequency activity (Fig. 6)^7,137–139^. Furthermore, power-power modulation of signals recorded from the pyramidal layer and dendritic layers is high in the broad gamma band, whereas ripple band activity in the pyramidal layer has a negative correlation with gamma power^25,76,77,134^ (Fig. 6^140^). While these experiments clarify the physiological distinction between SPW-Rs and gamma oscillations in the ‘traditional’ gamma band (30–120 Hz), the relationship with higher frequency ‘gamma’ and broadband ‘gamma’ needs further clarification.  A practical solution is to systematically quantify the power changes and cross-frequency power-power correlation for successful vs. unsuccessful recall trials by frequency band. A narrow peak in the 80–120 Hz band would favor SPW-R interpretation, whereas a broadband change or phase coupling to the theta oscillation would support the presence of high gamma oscillations or broadband gamma activity.

## Problem 6. SPW-Rs and pathological ripples

### Memory and seizure networks often overlap in the hippocampus

A final obstacle in working with human intracranial EEG data is the unique challenge of recording from the brains of patients with epilepsy. Seizure networks affect not only the seizure onset zone, but often involve widespread cortical networks, causing multi-domain cognitive deficits and structural, metabolic, and neurophysiological changes^141^. The hippocampus is particularly vulnerable to pathological recruitment, because of high connectivity to multiple brain regions. Seizures, interictal epileptic discharges (IEDs), and pathological ripples (or p-ripples) easily hijack this existing functional network. Hippocampus and associated temporal lobe structures are frequently implanted with depth electrodes during surgical localization of epileptic foci in the human brain, even when the primary hypothesis is that seizures are initiated in the neocortex.

This placement strategy provides a privileged opportunity to record hippocampal SPW-Rs in humans, during awake cognitive processes and NREM sleep^107^. However, because of the high degree of overlap between memory and seizure networks, physiological and pathological events must be meticulously separated.

Because SPW-Rs and p-ripples are observed during the same brain states and share overlapping mechanisms^6,54^, distinguishing between events is a formidable task. During SPW-Rs, a large fraction of neurons in the hippocampal-entorhinal system fire in synchrony with high excitatory gain. Because of their super-synchronous nature, even minor perturbations of the hippocampal circuits can turn SPW-Rs into high-frequency oscillations with more strongly synchronized population spikes, referred to as pathological or p-ripples^64,142–144^. Like the SPW-R complex, p-ripples can occur in isolation or ride on interictal epileptiform discharges (IED)^145,146^. Indeed, many IEDs in the hippocampus may be considered “exaggerated SPW-Rs” because their depth profiles are often identical to SPW-Rs initiated in the CA3 and CA2 regions, respectively^33^.

### Distinguishing between physiological and pathological ripples

The use of thresholds on low-level features such as frequency, duration, or amplitude can be problematic in a dataset containing both physiological and pathological events. While some have proposed non-overlapping frequency bands to distinguish between physiological ripples (80–250 Hz) and pathological ripples (“p-ripples”; 250-500 Hz)^121^, neither amplitude^147^ nor frequency range can reliably separate physiological SPW-Rs from p-ripples^147^. P-ripples can possess broadband peaks overlapping with the physiological ripple band (80–200 Hz) but analysis of the statistical distribution of p-ripples discloses strong spectral variability leaking into both the high and the low-frequency band in both rodents^54^ and humans^148,149^.

**Fig. 7:**
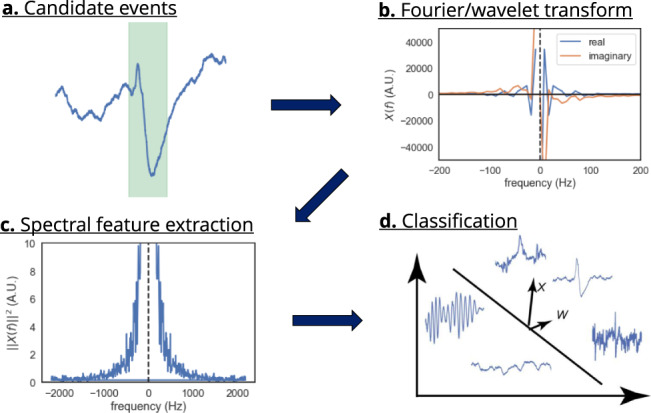
A machine learning approach to pattern classification. **a** The recording is segmented into (possibly overlapping) snippets short enough to contain at most one event. **b** Fourier transform of the event. **c** Spectral features extracted from Fourier/wavelet transform, followed by postprocessing steps. **d** Clustering is performed on the resulting features. Figure courtesy of Zhenrui Liao.

Yet, several criteria can improve the separation of SPW-Rs from p-ripples. P-ripples are more abundant in the primary epileptogenic zone and are typically unilateral^150^. Thus, exclusion of electrodes residing in the epileptogenic zone reduces the risk of p-ripple detection^39,121,151^, although it is understood that both interictal epileptiform discharges (IEDs) and p-ripples can occur throughout the epileptic brain^39,141^. Removing trials with IEDs (often with overriding p-ripple activity) further reduces the risk of contamination^39^. Several IED detection algorithms have been published with varying degrees of sensitivity and specificity^152–155^. P-ripples show wide variability in frequency (50 to 500 Hz), amplitude, and duration^69,156^. While events faster than 180 Hz can safely be categorized as p-ripples in^157^ (but see ref. 121), slower and low amplitude p-ripples are more difficult to be separated from SPW-R. Thus, spectral variability could be used to identify p-ripples (Fig. 7^54,148^). Finally, SPW-R and pathological high-frequency oscillations (HFO) may be separated during NREM sleep based on their differing phase relationship with slow-wave activity^158,159^. However, the tradeoff for stricter inclusion criteria is data loss.

Combining automated detection of both SPW-Rs and p-ripples with expert validation can be more effective than a simple thresholding approach. However imperfect, these automated detection algorithms have the benefit of applying objective criteria across datasets and laboratories, in contrast to subjective judgments by human operators^12,111,158^.

As is the case with SPW-R detection, machine learning techniques have also been applied to identify p-ripples, and IEDs^54,160–162^. Spectral approaches use wavelet transforms and time-frequency plots to capture all morphological event features^163,164^. Such inputs enable the downstream algorithm to learn their morphological features. This method approximates the human classification strategy by considering the shape of events while maintaining reproducibility between groups. Furthermore, including spectral features of the background would add information about the arousal state to this unsupervised model, and potentially better discriminate between fast oscillations. The performance of machine learning techniques against simpler feature-driven approaches should be tested in future experiments (Fig. 7^165^).

## Conclusion and recommendations

As translational discovery on the role of hippocampal SPW-Rs in human cognition gains momentum, we are confronted with varied approaches to recording, detection, and reporting methods. These differing techniques may explain much of the variance in reported results. The conundrum is that high-frequency events with a similar appearance (i.e., ripples and gamma oscillations) result from differing mechanisms and brain states. Conversely, shared mechanisms and brain states can drive high-frequency oscillations with different appearances (i.e., SPW-Rs and p-ripples). While confident separation and identification of high-frequency events must wrestle with the problems we have outlined, we argue that shared detection and reporting standards will improve confidence in findings and facilitate cross-species comparisons.

In experimental animals, the ideal list of physiological criteria to identify hippocampal SPW-Rs is shared across laboratories. Yet, methods of recording, analysis, and reporting still vary widely across and within laboratories. While the term SPW-Rs refers to discrete network events, they are embedded in perpetually changing brain dynamics with no clear boundaries. SPW-Rs exist on a wide continuum of amplitudes and durations and are separated from other events by imperfect threshold criteria. Most of the following recommendations are intended for human experiments, although some are appropriate for animal researchers as well.

### Experimental design and recording (humans)

There are many recommendations to improve SPW-R detection and identification in human experiments. The first is to monitor brain states more rigorously. Given their anti-correlation with cholinergic tone and arousal state, SPW-R and gamma could be more confidently separated by monitoring physiological features such as pupil size, heart rate, and background EEG activity. We also recommend simultaneous monitoring of micro-LFP and macro-LFP in the hippocampus and recording neuronal spiking activity when possible. Novel electrode arrays^166^ will improve recordings at multiple levels of spatial resolution. Finally, electrode sites should be localized to the hippocampal subfield and layer level.

### Detection and confidence estimation (animals, humans)

Instead of approaching SPW-R detection with a pre-defined and arbitrary bandpass filter, we recommend first inspecting the broadband data recorded from the CA1 subfield and looking for endogenous narrow-band peaks in activity. Power spectral density analysis of the detected individual events should reveal a significant narrow peak in the SPW-R frequency band, riding on the broadband 1/f frequency–power distribution. Calculating and reporting spectra from wider temporal windows, would also characterize the arousal state. Ideally, putative SPW-Rs detected during an experimental task should be compared with those found during NREM sleep. When unit recordings are available, SPW-R-unit histograms during both awake and NREM sleep should be compared. These recommendations also apply to p-ripples and neocortical ripples. Finally, confidence estimates on detected SPW-R events should be performed, with analysis performed on a subset of highly likely SPW-R events.

### Feature description (animals, humans)

Instead of reporting mean values, plotting the distribution of SPW-R features would enhance transparency and give insight into the degree to which identified events in different studies are comparable, providing important context to interpret similarities and differences. Examples of individual SPW-Rs, pathological activity and rejected artifacts should be graphically presented, preferably together with traces from nearby and more distal recording sites.

### Separation of physiological and pathological patterns (animals, humans)

Recording from the epileptic human brain and animal models of disease requires further special considerations. The reported hippocampal SPW-Rs in humans appear to be briefer than in rodents (Supplementary Table S1). Future studies are needed to clarify whether this difference is biological or results from different recording and detection criteria. An important control condition in humans would be the comparison to SPW-Rs (or just ripples) during NREM sleep. A caveat is that pathological events are also more abundant during NREM sleep. Anti-seizure medications may affect SPW-R occurrence and sleep. Further, as discussed above, the relationship between SPW-Rs, brain state, and theta/gamma oscillations requires further clarification.

### Reporting detection methods (animals, humans)

We recommend that published methods should detail detection criteria, including electrode types and sizes, precise localization of the electrode(s), filtering methods, type of filter(s), and the specific detection thresholds. For transparent interpretation, authors should report how their results manifest under a range of SPW-R detection parameters. If findings are similar across a range of parameters, the conclusions are more robust.

### Data and code sharing (animals, humans)

Public sharing of well-curated datasets would facilitate comparison of different detection methods and provide ‘ground truth’ material to develop automatic clustering methods. In datasets from closed-loop experiments, SPW-Rs detected and missed should be reanalyzed with offline methods, and report false positive and false negative rates. While errors are an inevitable consequence of any physiological analysis, error reporting will increase the confidence in findings.

The above recommendations will advance progress in SPW-R research. These recommendations could also apply to reliably identify neurophysiological events involved in cognition, sensorimotor behavior, and brain-machine interface applications. However, we recognize that ideal conditions are often not feasible due to cost and time and may not be crucial to every experiment. The proposed solutions are recommendations and not mandates. Progress is a community effort—dependent on the voluntary adoption of shared guidelines and transparency.

## Supplementary information

Supplementary Table 1
