# Supplementary material for: A consensus statement on detection of hippocampal sharp wave ripples and differentiation from other fast oscillations
Source: Nat Commun. 2022 Oct 12;13:6000. doi: 10.1038/s41467-022-33536-x (PMC9556539; doi:10.1038/s41467-022-33536-x)
Supplement: Supplementary file 1 — Supplementary Table 1 [file 41467_2022_33536_MOESM1_ESM.pdf]

Supplementary Table 1
RODENT METHODS

**Methods**	1	2	3	4	5	6	7	8
Running Speed (Threshold)	<5 cm/sec<br>for > 1 sec	n/a	n/a	n/a	<4 cm/sec	<5 cm/sec	n/a	n/a
Behavioral Tasks	Memory demanding tasks<br><br>Non-memory demanding tasks<br><br>Optogenetic manipulation of ripples	Slow-wave sleep	Awake, head-fixed mice on a cued treadmill or non-cued floating styrofoam ball	Slow-wave sleep	Freely moving conditions, NREM sleep	Linear track, Open-field exploration, goal-directed navigation	Linear track	Object recognition tasks, Freely moving conditions
Hippocampal Electrode Type and Location	Silicon probes in CA1 pyramidal layer	Tungsten wire in prefrontal cortex, motor cortex, thalamus, and hippocampus	Silicon probes, bipolar tungsten wire, glass	Tetrodes in MPFC and CA1	CA1 tetrodes	CA1 tetrodes	Teflon-coated steel wire	Silicon probes, glass pipettes
Reference	Screw electrode in the skull above the cerebellum	Ground and reference electrodes were positioned above the cerebellum	Silver/chloride wire in saline bath for acute recordings with silicon probes and glass. Chronic tungsten recordings are referenced to a superficial wire in callosum.	Stainless steel screws (reference and ground) were implanted above the cerebellum	Callosal	Bone screw	contralateral frontal bone	Occipital screw or silver/chloride; platinum/iridium wire for glass
Artifact rejection (EMG, Muscle)	CSD of multilayer recordings	Excluding motion artifacts and sleep spindles	Threshold ripple amplitude in cortical channels.	n/a	n/a	n/a	noisy LFP epochs removed	No a priori artifact rejection
High frequency Oscillation (threshold?)	Ripple: 80-300 Hz	100-500 Hz	90-200 Hz	<300 Hz	n/a	100-250 Hz<br>150-250 Hz	n/a	>100 Hz
Ripple Filter Band	80-250 Hz<br><br>Difference-of-Gaussians, zero-lag, linear phase FIR	80-250 Hz	Butter bandpass filter (90-200Hz)	100-250 Hz, then Subtract HF power (300-500 Hz)	150-250 Hz	Notch (60 Hz, 120 Hz, 180 Hz)	Down-sampled to 1250 Hz<br>Notch (60 Hz)<br>1/f correction	Butter filter (100-300 Hz passband for physiological; 100-600 Hz for pathological) & a non-casual filter
Ripple Amplitude	4 SD exceeding mean and SD of	3-5 SD	>5 SD	> 3 SD	>2 SD	3 SD, 5 SD	n/a, SPW-R identified via	>2-3SD

	baseline LFP during NREM sleep						wavelet-derived feature classification	
Ripple Duration	>15 ms	20-100 ms	>2 SD for 20 ms	> 1 SD for 30-110 ms	>15 ms	No limit or > 2 SD for 50 – 200 ms	n/a (1 calcium imaging frame)	>15 ms
Sharp Wave Detection	Yes. 8-40 Hz exceeding 2.5 SD of background signal	n/a	n/a	n/a	No	No	Yes – pathological (IED)	yes
Other:		Ripple and HFO detection using using RippleLab (Navarrete et el., 2016)	Offline graphical user interface (GUI) for event-by-event examination.				Semi-supervised classification using wavelet derived SPW-R features	Sharp-waves at SR are detected independently and paired to ripple detection at SP
Representative Publication	Fernandez-Ruiz et al. Science 2019	Li et al., Epilepsia, 2018	Varga et al. Elife 2014	Todorova & Zugaro, Science, 2019	Kay et al, Nature 2016	Pfeiffer & Foster, Nature, 2013	Sparks, Liao et al. Nat Comms 2020	Valero et al., Neuron 2017

n/a = not available

PRIMATE METHODS

Methods	1	2	3	4	5	6	7	8	9
Behavioral State	Sleep; we only observe unambiguous SW-R during rest	Episodic-like memory retrieval, visual search, and free-moving exploration of the environment. Quiet wakefulness and NREM sleep.	Retrieval of visual episodic memory and LT autobiographical memories and semantic facts	Awake, Memory retrieval	NREM Sleep	NREM Sleep	Awake Encoding and Retrieval of association task	NREM Sleep	Delayed free recall (categorized and uncategorized)
Electrode Type	Chronically implanted hyperdrive; 124 independently movable microwires (Tungsten, appx 100um)	Chronically implanted probes: TRec Pt/W tetrodes, microwire electrodes, linear arrays (16-128ch each).	Macroelectrode	Macroelectrode, Microelectrode, Single unit	Macroelectrode	Macroelectrode Only use channels with >100 detected events.	Macroelectrode	Macroelectrod e	Macroelectrode
Electrode Location	CA3, CA1, DG, Subiculum, Presubiculum, Parasubiculum , EC	CA3/DG, CA1, (pre/para)subi culum, EC, RSC	Within 2 mm from CA1, CA2/3, Subiculum	MTL/ MTG in Anterior Temporal Lobe	Hippocampus	Hippocampus	Anterior hippocampus	Different brain regions not affected by epilepsy	CA1, DG, entorhinal cortex, and parahippocampal cortex
Electrode Montage	Referential	N/A	Referential	Bipolar	Referential	Referential	Bipolar	Bipolar	Bipolar
Reference	titanium chamber with distributed contact across wide swath of skull/soft tissue; post-hoc "referencing" via ICA	Nearby depth reference microwire or titanium implant as "animal ground"	Subtract nearby WM reference signal	System reference hard wired as clinical contact	Linked Mastoids	Subtract nearby WM reference signal	Inverted strip electrode at site of craniotomy	Skull screw remote from epileptic region	System reference hard wired as clinical contact
Frequency Band	95-180Hz	100-250 Hz	70-180Hz	80-120 Hz	80-120 Hz	80-150 Hz	80-120	80-250 Hz	70-178 Hz (80-120 and 125-200 Hz in Supplemental)
Duration	>25 ms	>50ms	20-200 ms	>25 ms	>38 ms <500 ms	20-100 ms	20-200 ms	> 4 oscillations +effective duration of filter response	20-200 ms
Amplitude	>4 SD normal dist or >0.995	>3 SD crossing and at least 50ms >1 SD	Candidate events: >4 SD	>2 SD for detection, > 3 SD	>2.5 SD <9 SD Of mean	>2 SD for detection, > 5 SD	>2 SD for detection, > 4 SD	>3 SD	Candidate events: >4 SD

	CDF of Gamma dist	plus rejection of common (artifact) events	Expanded events: 2 SD	max amplitude for inclusion		max amplitude for inclusion	max amplitude for inclusion		Expanded events: 2 SD
Artifact rejection (including 60 Hz, muscle)	Relative amplitude in ripple band must be 2X of adjacent bands (70-90; 185-250) to eliminate broadband noise (like chewing). Cross-channel identical phase (from hilbert transform of ripple band filtered signal) for the duration of event (each channel is minimum 1.5mm away)	As #3 rodent: out-of-layer/neocortical signals must not show ripple-band activity (logical 'NOT') including subthreshold cross correlation or zero-phase coherence.	Use the common average iEEG signal, computed across all electrodes to exclude ripple-like muscular/electrical artifacts.<br>60, 120, 180Hz notch filters.	60 Hz Notch filter, remove noisy trials that exhibit excessive kurtosis or variance	50 Hz (and harmonics up to 200Hz) Notch filter; data marked as artifact if z-scored amplitude, z-scored amplitude gradient, or z-scored high-frequency amplitude (>250 Hz) exceeded 5 *or* if the conjunction of the three measures exceeded 3.	Unnecessary during NREM sleep	60 Hz Notch, Noisy trials removed via visual inspection	Unnecessary during NREM sleep	58-62 Hz notch, channels <0.1 Hz ripple rate removed. Automated detection for correlations across channels and within each channel across trials.
IEDs	n/a	n/a	25-60 Hz detecting transient events >4 SD	>250 Hz; First derivative or high pass filtered signal (>250 Hz) with a z-score > 5 marked as artifact/IED - removed time points -100 to 100 ms around event.	>250Hz IED	z-scored amplitude or derivative >5 SD	Automated IED detection via statistical distribution of iEEG voltages (Janca, 2014)	Electrodes with IEDs any time of the circardian cycle were excluded	25-60 Hz (>250 Hz in Supplemental using Vaz algorithm)
SOZ	n/a	n/a	Validation analysis: results do not change when excluding ripples that coincide with typical pathological HFOs.	Not considered	Electrodes in primary SOZ excluded	Electrodes in primary SOZ excluded	Electrodes in primary SOZ excluded	Excluded	Compare SOZ and non-SOZ electrodes in Supplemental

Other	Adjacent events <20 ms separation merged.		Adjacent events <30 ms separation merged<br>Ripple-band power was computed by clipping extreme values to 4 SD. Extreme values were defined using the Least-Median-Squares (LMS) method.<br>The threshold for ripple detection (4 SD of mean), was defined based on the clipped signal to minimize ripple-rate induced biasing.		>3 cycles in raw signal; discrete peak between 80 and 120 Hz in spectrogram			Adjacent events <20 ms separation merged.	Same details as Norman et al (3rd column)
Representative Publication	J. W. RUECKEMANN, A. DEDE, Y. BROWNING, E. A. BUFFALO, Traveling sharp-wave ripples in the monkey hippocampus. SFN 2019.	Leonard & Hoffman., Current Biology, 2016	Norman et al., Neuron, 2021	Vaz et al., Science, 2019	Staresina et al., Nature Neuroscience, 2015	Skelkin et al., PNAS, 2021	Henin & Liu, Brain, 2020	Frauscher et al., Annals of Neurology, 2018	Sakon & Kahana, bioRxiv, 2021
